# Extent of lymphadenectomy in radical cystectomy for bladder cancer

**DOI:** 10.1186/1477-7819-3-43

**Published:** 2005-07-15

**Authors:** M Hammad Ather, Sadaf Fatima, Orhun Sinanoglu

**Affiliations:** 1 Department of Surgery, Aga Khan University Hospital, Karachi, Pakistan; 2 Taksim Teaching Hospital Urology Clinic, Istanbul, Turkey

## Abstract

**Background:**

The benefit of pelvic lymphadenectomy in patients with cancer of the urinary bladder remains controversial. Though the inclusion of lymph node dissection in conjunction with radical cystectomy for patients with clinically negative nodes is well accepted, however, the extent of the nodal dissection remains contentious, particularly in patients with gross disease and T_1_G_3 _cancer. The extent of the primary bladder tumor, number of lymph nodes removed and the lymph node tumor burden are important prognostic variables in patients undergoing cystectomy. We analyzed the impact of the extent of lymphadenectomy during radical cystectomy on survival in the contemporary literature.

**Methods:**

A Pubmed search was carried out for the literature published over the last 15 years using bladder cancer, radical cystectomy, survival, lymphadenectomy and complications as the key words. We have discussed the extent of lymphadenectomy on survival and its anatomical basis to determine the optimal number of lymph nodes to be removed and the concept of node density.

**Results:**

Evidence from contemporary literature indicate significantly increased survival rates after cystectomy in patients with bladder cancer diagnosed with stages III or IV disease who have had relatively more lymph nodes examined, suggesting that even some patients with higher stage disease may benefit from extended pelvic lymphadenectomy at the time of cystectomy. Studies also indicate that more extensive lymphadenectomy significantly improved the prognosis of patients with bladder cancer, not only by providing prognostic information but perhaps it is also due to its inherent therapeutic value.

**Conclusion:**

Extended lymph node dissection improves local control and survival. However, in the absence of controlled randomized trial this remains a dubitable issue.

## Background

Bladder cancer is the second most common tumor of the urogenital tract with transitional cell carcinoma comprising of about 90% of all primary bladder malignancies [1]. Most of these tumors are superficial at initial presentation, limited to the mucosa, sub-mucosa or lamina propria. In superficial cancers, recurrence rates after initial treatment are 50–80% with progression to muscle invasive disease in 10–25% cases, whereas in muscle invasive disease there is 50% risk of loco-regional and distant metastasis.

The transitional cell carcinomas (TCC) originates in the bladder mucosa, progressively invading the lamina propria and more sequentially involves muscularis propria, perivesical fat and contiguous pelvic structures with increasing incidence of lymph node involvement during progression [2,3].

Radical cystectomy is the corner stone of treatment for invasive bladder cancers in patients whose medical condition allows major surgical procedure [1,4]. Five-year life expectancy is 75% for T_2 _lesions, 40% for T_3 _and 25% for T_4 _lesions [5]. The outcome for bladder cancer patients have improved primarily because of advances in surgical technique and better peri-operative care. However 70% of the patients with node positive disease develop tumor recurrence if treated by cystectomy alone. Proper staging of disease and timely institution of adjuvant treatment could potentially make a difference.

In addition to the evaluation of the primary tumor in a cystectomy specimen, assessment of the regional lymph nodes is also important; not only to accurately stage the disease but also to identify the need for adjuvant treatment. Preoperative imaging studies CT scan or an MRI will miss microscopic nodal metastasis in up to 70% of the patients [3,7,8] and this leaves histological evaluation as the only reliable tool in accurately staging the disease. The reported incidence of regional lymph node involvement following radical cystectomy is between 14% and 28% [[5,6], and [8]]. These rates correlate with the stage of the tumor; the higher the stage, the higher is the incidence of the involved lymph nodes [5,10,11]. Lymph node involvement is associated with increased risk of local recurrence and disease progression with survival rates varying from 20% to 40% in patients with and without lymph nodes metastasis respectively [5,10].

Pelvic lymphadenectomy is routinely performed as part of radical cystectomy for bladder cancers; however, there is lack of consensus on the intent (therapeutic or diagnostic) and limits of lymph node dissection. It is most often performed as a staging procedure. Some of the leading authorities believe that it has a potential therapeutic adjunct to radical cystectomy [12,13].

The absolute limits or extent of lymphadenectomy for patients undergoing surgery with a curative intent has not been precisely defined. The recent guidelines for the treatment of muscle invasive bladder cancer by The European Association of Urology recommend limited pelvic node dissection. This consists of removing the tissue in the obturator fossa in patients undergoing surgery with a curative intent [14]. Whereas several authors have noted an improved 5-year survival with extensive pelvic lymph node dissection in patients with node positive bladder cancer [2,3,15].

In the present review, we have attempted to analyze the current literature to see if there is convincing data to support the observation of some authors that extensive pelvic lymphadenectomy is associated with low pelvic and distant recurrence and survival.

## Extent of lymphadenectomy

The minimum number of lymph nodes to be excised in order to obtain a therapeutic advantage remains an issue of controversy. Anatomical studies have defined the external and internal iliac lymph nodes and the obturator nodes as the site of primary lymphatic drainage of the bladder and the common iliac nodes as the site of secondary drainage, with the trigone and the posterior wall draining directly into the pre-sacral nodes [16].

The role and extent of lymphadenectomy in bladder cancer is not well-defined, in contrast, patients with gastric [17], breast [18], and colorectal [19] tumors the impact of lymphadenectomy has been well studied and there has been a consensus that complete lymph node dissection with removal of a minimum number of lymph nodes correlates with improved survival. Guidelines for the management of colon and rectal cancer indicate that at least 12–14 lymph nodes should be retrieved by the pathologist in patients with colorectal cancers [18] and at least 10 lymph nodes in patients with endometrial cancer [20], but this knowledge is lacking in cases of bladder cancers.

Several authors have postulated that the number of lymph nodes examined in cystectomy specimen can have an impact on the outcome of patients treated for primary bladder cancer [9,13], whereas others have reported an improved survival with extensive nodal dissection [15,22]. Extensive lymphadenectomy increases the operating time and theoretically may increase the risk of hemorrhage and lymphocele formation, but studies have not shown a significant increase in morbidity with this procedure [22].

## Number of lymph nodes and impact on survival

Many factors can influence the outcome of patients with bladder cancer including the T-stage, the N-stage and the total number of lymph nodes retrieved. Skinner *et al, *[13] noted that important prognostic factors included age, gender and lymph nodes status but the number of involved nodes was the single most important prognostic variable. Lerner *et Al*, [2] observed that the 5-year survival probability in cases with 5 or fewer involved nodes was almost double than those with 6 or more involved nodes. Mills *et Al*, [23] studied a group of 83 patients with nodes positive disease and reported similar results. The median number of retrieved nodes was 20/case. A significant survival difference was observed when patients with less than 5 involved nodes were compared with more than 5 involved lymph nodes (p = 0.0027), however this significance was lost on multivariate analysis. Stein *et al, *[22] verified the impact of positive nodes on univariate as well as multivariate analysis with a cut off of 8 involved nodes [18]. Vieweg *et al*, [24] reported that in cases with organ confined disease, there was no survival difference between N_0 _and N_1 _groups but survival benefit consistently decreased from N_1 _to N_3 _disease. Konety *et al, *[25] studied 20,799 patients treated for primary bladder cancer and reported similar results. They concluded that regardless of the tumor stage, at least 14 lymph nodes should be excised in patients undergoing radical cystectomy. Herr *et al, *[26] studied the impact of lymph nodes in a group of 322 patients who underwent pelvic node dissection. They observed that in patients with pN_0 _status improved survival and local control was seen when the cut-off was 8 examined lymph nodes. However, in patients with pathologically involved nodes at least 9 lymph nodes should be removed.

## Anatomical extent of dissection

Poulsen *et al*, [15] studied the influence of extent of nodal dissection on survival following radical cystectomy for bladder cancer. They compared 126 patients who underwent extensive pelvic dissection with proximal limits up to the bifurcation of aorta and 68 patients who underwent limited nodal dissection with proximal limits up to the common iliac vessels. The overall prevalence of nodal metastasis was slightly higher in patients with extended dissection as compared to ones with limited dissection. Extended dissection was associated with an improved 5-year recurrence free survival for patients with tumor confined to the bladder (pT_3a _or less), but this was not true for tumors penetrating the bladder wall. This was a retrospective, non-randomized study and the anatomical mapping and the number of retrieved lymph nodes was not mentioned.

Herr *et al*, [21] introduced the term of lymph node density indicating the ratio between the numbers of nodes removed to the number of involved node. They found ratio based lymph node staging, which reflects the quality of lymph node dissection, was a significant prognostic variable for survival and local control in patients with lymph node positive bladder cancer after radical cystectomy. Later Stein *et al*, [22] reported their experience of 244 patients with involved lymph nodes treated for primary transitional cell carcinoma of bladder. They reported that the overall and recurrence free survival was significantly related to pathological subgroup of the primary bladder tumor, patients with organ-confined disease having a better survival as compared to those with extravesical tumors. Patients with a lymph node density of 20% or less had a better recurrence free survival when compared to those with more than 20% (p < 0.001), however this study confined to only the lymph node positive cases, the lymphadenectomy was done in *en bloc *fashion, and the exact anatomical location of the excised lymph nodes was not known. In an attempt to standardize the extent of dissection, Leissner *et al *[9] studied a group of 447 patients who underwent extended lymphadenectomy along with radical cystectomy for bladder cancer. The guidelines for lymphadenectomy were to remove obturator, internal, external and common iliac, pre-sacral and the lymph nodes on both sides of aortic bifurcation. The mean number of retrieved nodes was 16.7/patient. They observed that overall the tumor specific survival (P-value < 0.013) and disease free survival was significant (P-value <0.016) when patients with ≥ 16 excised lymph nodes were compared with those with < 15 nodes removed, except for those with T-4 lesions. In presence of nodal metastasis patients only benefited by removal of at least five metastatic lymph nodes, thus supporting the therapeutic effect of extended lymphadenectomy.

The two possible reasons for better survival in patients with extended lymphadenectomy were explained by lymphadenectomy resulting in a more accurate staging so adjuvant therapy may be started earlier. The other possible explanation could be the increased likelihood of removing nodal tumor deposits. Conclusive evidence could not be derived from a retrospective studies and literature review.

Mills *et al *[23] reported their experience of 83 patients with node positive tumors who underwent pelvic lymphadenectomy. Anatomical mapping was done in the group of retrieved nodes. Survival correlated with the number of lymph nodes, adjuvant chemotherapy, capsular penetration and the site of metastatic nodes. On univariate analysis, survival was better when more than 5 positive lymph nodes was removed however this was not significant and only lymph node capsular penetration demonstrated a significant effect on survival. This was again a retrospective study but only the pelvic lymph nodes were dissected, therefore the nodal status of the proximal sites was unknown.

Vazina *et al *[27] reviewed their results of 176 patients who were treated with lymph node dissection up to the aortic bifurcation. The median number of lymph nodes excised was 25/case. It was observed that the rate of nodal involvement gradually decreased from pelvic to aortic sites. They reported that these tumors did not exhibit skip metastasis as all. The patients except one who had involvement of lymph nodes at or above the aortic bifurcation or common iliac region also had positive lymph nodes in distal (pelvic and peri-vesical) areas. One patient who had involvement of the common iliac lymph nodes without involving the distal lymph nodes, had a primary lesion in the trigone explaining this to be presumably due to direct lymphatic drainage of the trigone area to the common iliac region. Bochner *et al, *[28] reported the survival status of 144 patients who were operated for primary bladder cancer, out of whom 56 underwent standard pelvic node dissection whereas 88 patients had extended dissection. Though, the yield of nodes was greater in extended dissection than in standard dissection, but both types had a similar percentage of patients with positive lymph nodes, however extended lymphadenectomy provided better prognostic information through a more accurate evaluation of the total number of lymph nodes excised or the number of involved lymph nodes. Extended lymphadenectomy identified 33% of patients with microscopic nodal metastasis involving the common iliac lymph nodes, explaining the therapeutic value of pelvic dissection. This is also supported by a European study of a group of 290 patients, who underwent extended radical lymphadenectomy along with radical cystectomy [29]. In this series, it was observed that 57% of patients with lymph node involvement at level I had nodal metastasis at level II and 31% had level III nodes involved. Similarly, 35% patients with level II nodes involved were found to have level III nodal involvement, hence they concluded with a strong recommendation for extended lymphadenectomy in all patients undergoing radical cystectomy for bladder cancer with a curative intent. The three lymphadenectomy levels were defined by the authors as; level I, distal to the bifurcation of common iliac artery, level II distal aortic bifurcation up to level I and level III proximal to aortic bifurcation.

## Conclusion

More than half the patients with locally advanced (pT_3a, _pT_3b,_pT_4a _and/ or pN+) treated by radical surgery will have disease progression in five years. Adequate staging affects the outcome of patients with bladder cancer. Such information is important not only for therapy and prognosis, but also in identifying individuals for adjuvant treatment. Evidence from contemporary studies indicates that more extensive lymphadenectomy significantly improved the prognosis of patients with bladder cancer. A growing body of evidence suggests that an extended lymph node dissection may provide not only improved prognostic information, but also a clinically significant therapeutic benefit for both lymph node-positive and -negative patients undergoing radical cystectomy. However, in the absence of randomized controlled trial, the scientific value of current evidence is at best level 2.

## Competing interests

The author(s) declare that they have no competing interests.

## Authors' contributions

**MHA**. Conceived of the idea, helped in literature search and drafted part of the manuscript.

**SF **did the literature search, drafted most of the manuscript.

**OS **Wrote the initial draft and helped in literature search. All authors have read and approved of the final draft.
